# Assessing the Prevalent Myths and Misconceptions Among Caregivers of Patients With Cancer: A Cross-Sectional Study

**DOI:** 10.7759/cureus.51332

**Published:** 2023-12-30

**Authors:** Pallvi Kaul, Deepti Choudhary, Ajeet R Tiwari, Rythm Walia, Sanjay Sadhu, Pankaj K Garg

**Affiliations:** 1 Surgical Oncology, Shri Guru Ram Rai Institute of Medical and Health Sciences, Dehradun, IND; 2 Obstetrics and Gynaecology, Shri Guru Ram Rai Institute of Medical and Health Sciences, Dehradun, IND; 3 Biotechnology, Shri Guru Ram Rai University, Dehradun, IND; 4 Plastic Surgery, Shri Guru Ram Rai Institute of Medical and Health Sciences, Dehradun, IND

**Keywords:** cancer education, cancer awareness, caregivers, treatment delays, cancer myths

## Abstract

Introduction

Certain popular ideas about how cancer starts and spreads, though scientifically wrong, can seem to make sense, especially when those ideas are rooted in old theories. The present study was conducted to assess the prevalence of myths and misconceptions among caregivers of patients with cancer.

Materials and methods

A hospital-based survey in a tertiary teaching hospital in a sub-Himalayan region of North India was conducted where caregivers (aged 18-70 years) were administered questionaries containing 10 close-ended questions. The study was conducted in small batches of 20-25 participants. The questionnaire was analyzed, and a healthcare worker discussed it with the participants and clarified their myths.

Results

A total of 400 participants were included in the study. The median age of the participants was 45 years (IQR 35-59). The majority of the participants were males (85%, n=340). The prevalent myths among the caregivers were the following: (a) cancer is always very painful (45.5%), (b) the cause of cancers is some sin/harm done to others (26%), (c) cancer results from some form of injuries (22.8%), and (d) cancer spreads from one person to another (20.8%). Over 90% of the participants (347/378) informed that post-survey counselling was effective in ameliorating their myths.

Conclusion

The present study highlights the widespread cancer myths and misconceptions among the caregivers of patients with cancer. Therefore, the need of the hour is to eliminate them to avoid any unnecessary treatment delays and strengthen the emotional and social support system for patients with cancer.

## Introduction

The new global cancer data extracted from the GLOBOCAN 2020 database suggests that the annual global cancer burden has risen to 19.3 million cases and almost 10 million cancer deaths. The global cancer burden is expected to rise to about 28.4 million cases by 2040, a 47% rise from 2020, with a larger increase in developed (64% to 95%) versus developing (32% to 56%) countries due to demographic changes [1]. Cancer cases are projected to further increase by 81% in developing countries by 2030. These changes affect not only individuals but also threaten the economic development of communities and countries [2].

Many cancers are curable, provided they are detected early by screening and treated effectively. Cancer myths and misconceptions are potential barriers to early cancer diagnosis and treatment compliance. The social, emotional, and financial devastation that all too often accompanies a diagnosis of cancer is, in large part, due to the cultural myths and taboos surrounding the disease. Certain popular ideas about how cancer starts and spreads, though scientifically wrong, can seem to make sense, especially when those ideas are rooted in old theories. This can lead to needless worry and even hinder appropriate prevention and treatment decisions. Some of the common cancer myths include the notion that being diagnosed with cancer equates to a death sentence and that cancer is an individual's fate and not preventable. Preference for opting for alternative therapies not backed by adequate scientific evidence for the treatment of cancer and considering these strategies as free of any side effects is another common myth. This can lead to delays in seeking medical care, resulting in cancer progression and decreasing survival outcomes. Cancer myths can create fear and anxiety in patients, causing them to worry about the effectiveness of their treatment, the likelihood of survival, and the potential side effects of treatment. This can have a negative impact on their mental health and quality of life. Some cancer myths suggest that certain foods or diets can cure cancer or prevent it from recurring [3]. This can lead patients to adopt unnecessary dietary restrictions that may negatively affect their nutrition and overall health. Some cancer myths promote false hope and suggest that a cure for cancer is just around the corner. This can lead patients to become overly optimistic and pursue unproven or risky treatments that may do more harm than good. Cancer myths can promote the idea that cancer is caused by a personal shortcoming. This can lead to stigmatization and blame, causing patients to feel ashamed or guilty about their diagnosis. Other prevalent myths are that cancer is just a health issue, that it is a disease of the wealthy and elderly, and that it is prevalent in developed countries.

Undoubtedly, cancer is still a dreadful challenge for oncologists and researchers, but that does not stop us from combating this fatal disease. Therefore, the need is to emphasize debunking innumerable myths and misconceptions associated with cancer. The present study assesses the prevalence of myths and misconceptions among caregivers of cancer patients, as they play an important role in providing support, financial, emotional, social, and physical, during the patient's treatment journey [4].

The results of the study were previously posted to the Research Square preprint server on September 19, 2022.

## Materials and methods

This study was a cross-sectional survey based on questionnaires conducted in a tertiary teaching hospital in the sub-Himalayan region of North India. The sample size was determined using the formula n=z^2(p)(1−p)/d^2. Considering the 50% prevalence of cancer myths and misconceptions among caregivers of patients with cancer (p=0.05), the sample size (n) was calculated to be 384 with a 95% confidence level and 5% precision of estimate (d=0.05). The study focused on caregivers (aged 18-70 years) of patients with cancer. We opted to include caregivers instead of patients in the study, recognizing that a cancer diagnosis may lead to significant psychological distress and trauma for patients, potentially affecting their willingness to participate. Additionally, caregivers play a crucial role in the treatment of cancer patients, as mentioned earlier.

Informed consent was obtained from all participants before their involvement in the study. Participants were assured that their participation was entirely voluntary and would not impact the treatment of their patients. The absolute anonymity of responses was guaranteed during the survey.

The questionnaire was drafted by the authors based on their prior experiences communicating with caregivers of cancer patients and insights from three external oncologists. It comprised 10 closed-ended questions, available in two of the most commonly spoken languages in the country, Hindi and English. The questionnaire underwent pretesting on 10 intended study participants. To facilitate participation for those willing but lacking sufficient reading and/or writing skills, a language translator was provided to aid comprehension and questionnaire completion. To ensure absolute confidentiality, only age and gender information was collected, fostering confidence in participants to answer questions with complete honesty.

The study was conducted in small batches of 20-25 participants per week. After completing the questionnaire, a healthcare worker discussed its contents and clarified any misconceptions. Ethical clearance from the Shri Guru Ram Rai University Institutional Ethics Committee was obtained before the study commenced (approval number: SGRR/IEC/02/22).

## Results

A total of 400 participants were included in the study. The median age of the participants was 45 years (IQR 35-59). The majority of the participants were males (85%, n=340). The majority of the participants opted for questionnaires in the Hindi language (94%), followed by the English language. A language translator was needed for eight (2%) participants. The discussion rounds were attended by 378 (94.5%) participants. At the end of the discussion rounds, a survey was conducted to assess the effectiveness of the intervention. About 91.7% of participants reported that the discussion rounds were "effective,” while 6% found them to be “somewhat effective.” Table 1 displays the demographic details and post-intervention survey responses of the study participants.

**Table 1:** Demographic details and post-intervention survey responses of the participants

Parameter assessed	n (%)
Gender of respondents (n=400)	
Male	340 (85%)
Female	60 (15%)
Age (in years)	
Range	19-68
Median (IQR)	45 (35-59)
Preferred language (n=400)	
Hindi	377 (94%)
English	15 (3.8%)
Translator	8 (2%)
Discussion rounds (n=400)	
Attended	378 (94.5%)
Missed	22 (5.5%)
Post-intervention survey (n=378)	
Effective	347 (91.7%)
Somewhat effective	23 (6%)
Not effective	0 (0%)
Can’t say	8 (2.1%)

**Figure 1:**
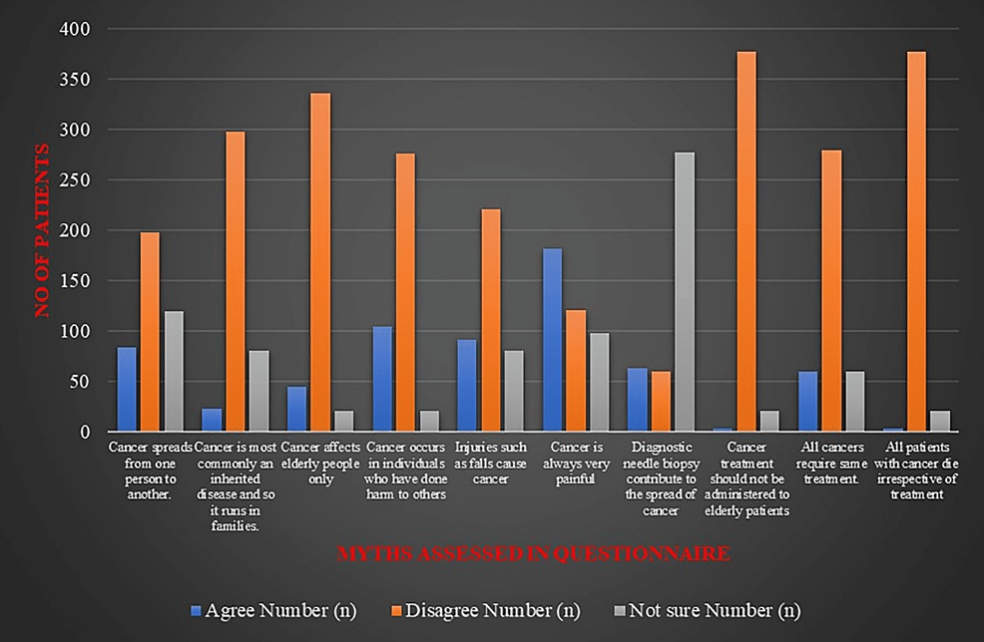
Histogram showing various responses of the participants to the cancer myths assessed in the questionnaire Cancer myths and misconceptions

The most prevalent myth among the caregivers was that cancer is always very painful (n=182, 45.5%), and only one-fourth (n=98, 24.5%) of the participants were sure that cancer may also be painless. Other common myths reported include cancer occurring due to some sin/harm done to others, cancer resulting from some form of injury, and cancer spreading from one person to another, reported by 26%, 22.8%, and 20.8% of the respondents, respectively. Diagnostic needle biopsy contributes to the spread of cancer, as reported by 15.8% of the respondents. The caregivers were also very concerned about the chances of inheriting the disease from the patients, especially if they were blood relatives. A majority of the participants (94.3%) in our study group disagree with the myth that all patients with cancer die irrespective of treatment. Figure 1 and Table 2 show the pictorial representation of the responses to the prevalent myths. Over 90% of the participants (347/378) informed me that post-survey counselling was effective in ameliorating their myths.

**Table 2:** Common myths and misconceptions prevalent among the caregivers of patients with cancer

Serial number	Myths	Agree		Disagree		Not sure	
		Number (n)	Percentage (%)	Number (n)	Percentage (%)	Number (n)	Percentage (%)
1	Cancer spreads from one person to another	83	20.8%	198	49.5%	119	29.8%
2	Cancer is most commonly an inherited disease and so it runs in families	22	5.5%	298	74.5%	80	20.0%
3	Cancer affects elderly people only	44	11.0%	336	84.0%	20	5.0%
4	Cancer occurs in individuals who have done harm to others	104	26.0%	276	69.0%	20	5.0%
5	Injuries such as falls cause cancer	91	22.8%	221	55.3%	80	20.0%
6	Cancer is always very painful	182	45.5%	120	30.0%	98	24.5%
7	Diagnostic needle biopsy contributes to the spread of cancer	63	15.8%	60	15.0%	277	69.3%
8	Cancer treatment should not be administered to elderly patients	3	0.8%	377	94.3%	20	5.0%
9	All cancers require the same treatment	60	15.0%	280	70.0%	60	15.0%
10	All patients with cancer die irrespective of treatment	3	0.8%	377	94.3%	20	5.0%

## Discussion

Culture is defined as a set of shared and socially transmitted ideas about the world that are passed down from generation to generation [5,6]. Culture as a socially transmitted phenomenon carries with it the idea that people who interact regularly know the same unwritten rules and criteria for social life that confer status as a member of the group [6]. When applied to illness, the beliefs and values from a cultural model of disease influence perceptions about the meaning of an illness, the useful types of treatment, and the likely outcome of health behaviours related to the prevention and control of the disease [7,8]. Cultural beliefs and values are increasingly being recognized as important determinants of not only attitude towards cancer prevention and control but also of psychological and behavioural outcomes following cancer diagnosis and treatment.

Cancer-related myths and stigma about cancer are important problems that must be addressed [9-11]. Cancer remains taboo, and people with cancer are even subjected to discrimination that may stop them from admitting that they have cancer. Many people perceive cancer to be a fatal disease. Fears about treatment can also fuel stigma. Certain beliefs, such as cancer, are contagious or a form of divine punishment for the sins committed in the past. Cancer symptoms or body parts affected by the disease can invoke stigma. Gynaecological or breast cancers may present with symptoms that women are reluctant to disclose to their doctors, and they may be even less willing to undergo the necessary physical examinations.

In the present study, the most prevalent myth among the caregivers was that cancer is always very painful, as reported by about 45.5% of the respondents. The majority of cancers in the early stages are usually painless, and early-presenting symptoms are usually ignored by the patients, resulting in a delay in seeking treatment or presentation at a relatively advanced stage, compromising the overall outcome of the treatment [12]. A diagnostic needle biopsy contributes to the spread of cancer, as reported by about 20% of the respondents. This myth was dispelled by the landmark study of more than 2,000 patients by Wallace et al. [13], which showed that patients receiving endoscopic ultrasound fine-needle aspiration (EUS-FNA) were marginally associated with improved overall survival without any impact on cancer-specific survival as compared to the non-EUS-FNA group. This study reinforced that physicians and patients should feel reassured that a biopsy in a cancer setting is a safe procedure. However, the contemporary literature does suggest that diagnostic needle biopsy is not recommended in a few resectable cancers, like renal cell carcinoma, for the risk of dissemination. However, we intended to assess the fear of diagnostic biopsy in the minds of cancer patients and their caregivers when it is indicated and recommended by the oncologists. Another common misconception about cancer in our study group was the fear of cancer being contagious, which was reported by about 20.8% of the respondents. These figures represent the tip of the iceberg, and the estimated prevalence of this myth far exceeds what is reported, especially in rural areas. Patients with cancer are often isolated by their family members, and avoiding sharing utensils and clothes or touching the patients is practiced. Unfortunately, several incidents of such practices are even reported by well-educated patients and their families [14]. This undermines the efforts to improve the quality of life of patients with cancer, which are challenged by discrimination, often based on unscientific and baseless assumptions. At the same time, it is important to create awareness regarding certain cancers associated with viruses that are transmitted by blood transfusion, shared needles, or unprotected sex [15]. A majority of the participants did not agree that all patients with cancer die irrespective of the treatment. This result might be slightly erroneous when extrapolated to the general masses, as our subset of participants includes caregivers and families who are well motivated to seek medical/expert opinion and proceed with the treatment in the best interest of their patients.

In 2008, the Union for International Cancer Control published the famous World Cancer Declaration. The fifth target of this declaration stated that "in 2020, public attitudes towards cancer will improve and damaging myths and misconceptions about the disease will be dispelled" about the importance of this issue. This, however, still seems like a far-fetched target.

Stigmas about cancer present significant challenges to cancer control. A negative public concept of cancer can perpetuate a cycle of fear and misinformation that hinders raising awareness about cancer prevention and the importance of early detection. Good practice requires an understanding of cultural and social aspects of life and death to enable care professionals to best meet the needs of patients and their families. Workforce education is important in the provision of culturally appropriate palliative and end-of-life care.

In a pilot survey conducted by Ray et al. [16] at the Chittaranjan National Cancer Institute, Kolkata, India, 900 people were assessed for their level of cancer awareness. The authors found that only 8% had prior exposure to any cancer awareness program: 37% on All India Radio, 36% on Doordarshan/private television channels, 34% via articles, and only 13% had seen cancer awareness posters and hoardings (unpublished findings). The results envisaged a great lacuna in cancer awareness prevailing within the public. Lack of awareness can be attributed as the root cause of the oncologic misconceptions. Cancer awareness programs can be a leading initiative and an effective tool to debunk these myths.

We believe that the simplicity of the study design and the requirement of minimal manpower to conduct the same permit easy replicability across various centres. An audit of our post-counselling sessions showed a positive trend and provided insight into incorporating this strategy as a transformative action for reformed attitudes to revolutionize the mindset to bring forth behavioural and systemic changes. Multicentric prospective cohort studies using validated questions specific to and of greater concern in a particular geographical area will give a better estimate of the prevailing burden of the pre-existing myths related to cancer and aid in devising an effective strategy to combat and debunk them.

We believe the following can be the potential strategies to ameliorate cancer myths among caregivers: (a) educating caregivers about the common cancer myths and misconceptions through educational materials or workshops that focus on debunking common cancer myths and misconceptions, (b) providing a safe space for caregivers and encouraging them to ask questions to dispel these myths, (c) providing access to reliable sources of information, such as reputable cancer organizations or government websites, to help caregivers get accurate information (else caregivers may turn to the internet or social media for cancer information, but these sources may promote misinformation or unproven treatments), and (d) involving caregivers in the treatment process. Caregivers who are involved in the patient's treatment process may have a better understanding of the disease and treatment options. Overall, providing accurate information, encouraging questions, and involving caregivers in the treatment process can help dispel cancer myths and misconceptions among caregivers. This can lead to better support for the patient and a better understanding of the realities of cancer.

The most important benefit that we derived from this study was that it provided us with a brief overview of some of the most prevalent myths in our geographic area. The information derived is being used by the oncologists at our centre to prospectively address these myths during patient counselling and inpatient visit sessions. This helps to ensure treatment compliance, decrease the chances of treatment abandonment, seek alternative treatment strategies that are not backed by concrete evidence, and improve the oncological outcomes of our patients.

Limitations

Our results may underestimate the percentage of the population having some common myth associated with cancer, as it represents the population motivated enough to seek medical intervention, contrary to the cohort of cancer patients who never seek an expert opinion or opt for alternative treatment options and succumb to the disease before reaching the oncologist. To keep the questionnaire crisp yet comprehensive, we may have underscored many other prevalent myths that might be more pertinent in some other geographic regions.

## Conclusions

The current study highlights that in the current era of digital health aiming at more efficient and sustainable health systems, healthcare professionals still need to tackle the lacuna of cancer awareness and widely prevalent myths and misconceptions associated with cancer, which are not only confined to low- or middle-income countries but are a global healthcare concern. These popular ideas about how cancer starts and spreads, though baseless and scientifically incorrect, can seem to make sense among the common masses, especially when these ideas are rooted in old theories. Appropriate communication via the health workers is a concept that needs urgent implementation to create a liaison between the health care providers and the community, resulting in early diagnosis, appropriate treatment, and ultimately improved outcomes.

## References

[1] Sung H, Ferlay J, Siegel RL, Laversanne M, Soerjomataram I, Jemal A, Bray F (2021). Global cancer statistics 2020: GLOBOCAN estimates of incidence and mortality worldwide for 36 cancers in 185 countries. CA Cancer J Clin.

[2] (2013). Cancer awareness campaigns: dispelling the myths. Lancet Oncol.

[3] Domínguez M, Sapiña L (2020). From sweeteners to cell phones-cancer myths and beliefs among journalism undergraduates. Eur J Cancer Care (Engl).

[4] Zhao XS, Gui L, Zhou LJ, Zhang B, Chen HY (2023). Risk factors associated with the comprehensive needs of cancer caregivers in China. Support Care Cancer.

[5] Koltko-Rivera ME (2004). The psychology of worldviews. Rev Gen Psychol.

[6] (1992). Human motives and cultural models.

[7] Coreil J, Wilke J, Pintado I (2004). Cultural models of illness and recovery in breast cancer support groups. Qual Health Res.

[8] Nierkens V, Hartman MA, Nicolaou M (2013). Effectiveness of cultural adaptations of interventions aimed at smoking cessation, diet, and/or physical activity in ethnic minorities. a systematic review. PLoS One.

[9] Link BG, Phelan JC (2006). Stigma and its public health implications. Lancet.

[10] Keusch GT, Wilentz J, Kleinman A (2006). Stigma and global health: developing a research agenda. Lancet.

[11] Kaul P, Kumar R, Singh MP, Garg PK (2021). Social taboos: a formidable challenge in cancer care. BMJ Case Rep.

[12] Hanna TP, King WD, Thibodeau S (2020). Mortality due to cancer treatment delay: systematic review and meta-analysis. BMJ.

[13] Ngamruengphong S, Swanson KM, Shah ND, Wallace MB (2015). Preoperative endoscopic ultrasound-guided fine needle aspiration does not impair survival of patients with resected pancreatic cancer. Gut.

[14] Garg PK (2015). What plagues cancer: does it spread?. Oncologist.

[15] Biswas J (2014). Debunk the myths: oncologic misconceptions. Indian J Med Res.

[16] Ray K, Mandal S (2004). Knowledge about cancer in West Bengal - a pilot survey. Asian Pac J Cancer Prev.

